# Clinical Reliability and Diagnostic Value of Digital Rectal Examination in the Detection of Prostate Cancer and Broader Clinical Practice: A Narrative Review

**DOI:** 10.7759/cureus.96832

**Published:** 2025-11-14

**Authors:** Amgad Elmadani, Oluseyi H Ogunfusika, Tagelsir Saeed

**Affiliations:** 1 Urology, Kettering General Hospital NHS Trust, Kettering, GBR

**Keywords:** 2-week-wait referral, diagnostic test accuracy, digital rectal examination (dre), multiparametric mri (mpmri), prostate biopsy, prostate cancer (pc), prostate-specific antigen (psa), urology clinic, urology imaging, uro oncology

## Abstract

Digital rectal examination (DRE) continues to occupy a central role in the assessment of urological disease, providing a quick, inexpensive, and simple method to evaluate the prostate gland as well as the surrounding pelvic structures. Although the diagnostic landscape has changed somewhat due to the increasing use of serum prostate-specific antigen (PSA) testing, multiparametric MRI, and molecular biomarkers, "classic" DRE retains an important clinical, educational, and ethical value in many areas of medical practice. Before the establishment of PSA screening and advanced imaging, DRE was the predominant method for detecting abnormalities in the prostate. It is not very sensitive for early disease detection, but it is priceless in providing a tactile examination of prostate dimensions, texture, and consistency used to differentiate benign prostatic enlargement, inflammation, or malignancy. Although not currently recommended for population screening in asymptomatic men, DRE continues to play an important role in assessing symptoms, triaging patients, and staging disease within individualised diagnostic pathways. In daily urological practice, it aids in the evaluation of patients with lower urinary tract symptoms (LUTS), including acute urinary retention and pelvic pain syndromes, and helps resolve uncertainty regarding borderline PSA levels or equivocal imaging results. Outside urology, DRE has wider clinical applications for assessing anal tone and perianal sensation in conditions such as cauda equina syndrome, evaluating rectal bleeding or palpable masses within coloproctology practice, and post-operative or emergency surgical review. Ethical conduct of the examination requires clinicians to communicate sensitively, obtain consent, and offer a chaperone so as to preserve patient dignity and trust (principles now firmly within professional behaviour and medical teaching). Recent developments in medical education have breathed new life into DRE training towards the implementation of simulation technology, haptic demonstration models, and augmented reality programmes which allow for standardised feedback and objective assessment of competence to counter past barriers centred around patient discomfort, variable supervision, and trainee self-assurance. Although contemporary diagnostic paradigms have shifted to PSA-based, image-directed, and biomarker-associated methods, DRE is indispensable because of the ease of use, low cost, and universality. Its continuing presence in national and international guidelines reflects its persistent applicability as a patient-centred, evidence-based procedure that also serves to bridge the gulf between traditional clinical acumen and state-of-the-art diagnostic accuracy.

## Introduction and background

Prostate cancer is one of the most common diseases in men and one of the world's leading causes of cancer-related mortality. A total of nearly 1.47 million new cases and 397,000 deaths were estimated worldwide by GLOBOCAN 2022, representing around 15% of all male cancers [1]. The incidence rates are extremely diverse, with the highest in Northern and Western Europe, North America, Oceania and lowest in Asia as well as sub-Saharan Africa [1,2]. These discrepancies are due to variation in PSA-testing uptake, life expectancy, quality of the healthcare system, and registry [2].

In the United Kingdom, prostate cancer is the most common cancer in men, with over 52,000 new cases and more than 12,000 deaths per year [3]. The five-year survival rate already exceeds 85%, mainly due to earlier diagnosis and improvements in the surgical treatment modalities, radiotherapy, and chemotherapy [3,4]. Despite these developments, however, prostate cancer remains responsible for approximately 7% of all male cancer deaths in England and Wales [3].

Historical development of prostate assessment

Before the availability of PSA testing in the late 1980s, DRE in male patients was the main method that urologists used to assess the prostate. Irregularity, nodularity, or hardness on palpation was suggestive of malignancy, yet no finger can palpate more than the posterior and lateral surfaces of the gland [5]. Jewett’s landmark 1952 series on performing DRE demonstrated that most of the cancers detected by this method were advanced and often extracapsular [6].

Early detection was revolutionised by the advent of serum PSA. Catalona et al. (1991) also found that PSA screening detected smaller, organ-confined tumours suitable for curative therapy [7]. A subsequent multi-centre comparison demonstrated that PSA combined with DRE increased detection modestly over either alone [8]. Two epic trials, the European Randomised Study of Screening for Prostate Cancer (ERSPC) and the Prostate, Lung, Colorectal and Ovarian (PLCO) cancer-screening trial, have set the modern benchmark. ERSPC reported a 21% prostate-cancer-mortality reduction with PSA-based screening [9], and PLCO showed no mortality difference overall, but high opportunistic PSA testing in the control group [10]. In the PLCO trial, less than 3% of screen-detected cancers were detected by DRE only [10].

Declining diagnostic value of DRE

Despite detecting tumours overlooked by PSA, DRE lacks high diagnostic accuracy. Pooled sensitivities and specificities are 0.51 and 0.59 for a meta-analysis in primary-care settings, from 2018 [11]. A subsequent European study found that DRE contributed little to diagnosis when combined with PSA in the modern era in present-day populations [12]. As a result, contemporary recommendations, including the European Association of Urology (EAU) 2024 [13], American Urological Association/Society of Urologic Oncology (AUA/SUO) 2023 [14], and National Institute for Health and Care Excellence (NICE) NG12 (2025) [15], recommend against performing routine DRE in asymptomatic men, instead recommending it for symptomatic evaluation and risk stratification.

Persisting clinical relevance

Although DRE's screening value has decreased, it is indispensable in daily urology practice. In men suffering from lower urinary tract symptoms (LUTS), DRE serves to estimate prostate size and consistency of the organ and its tenderness; an aspect critically important when differentiating BPH, prostatitis, and malignancy [16,17]. A fair correlation between DRE-estimated and ultra-sonographic gland volume have been found in some studies, suggesting its utility at the bedside [18]. DRE findings also direct therapy of AUR, acute or chronic prostatitis, and pelvic-pain syndromes [19,20].

Apart from clinical work, DRE also has an important learning and competence value. Simulation-driven programmes using models or manikins increase trainee confidence and reproducibility [21,22]. Digital rectal examination remains a fundamental clinical skill that all medical students are expected to learn and perform competently; however, confidence and formal training opportunities are often limited, prompting the need for structured teaching interventions [23].

Finally, DRE offers a lot of important data not only for the prostate. In the neurologic and colorectal fields, it is still a mandatory exam to assess anal tone, rectal masses, and signs of cauda equina syndrome [24]. It is also useful for quick cancer diagnosis and urgent referral in the framework of NHS urgent referral pathways [25].

Therefore, although its mass-screening function has declined, the DRE is an important part of general physical examination and urological work-up.

## Review

Methodology and literature search

**Figure 1:**
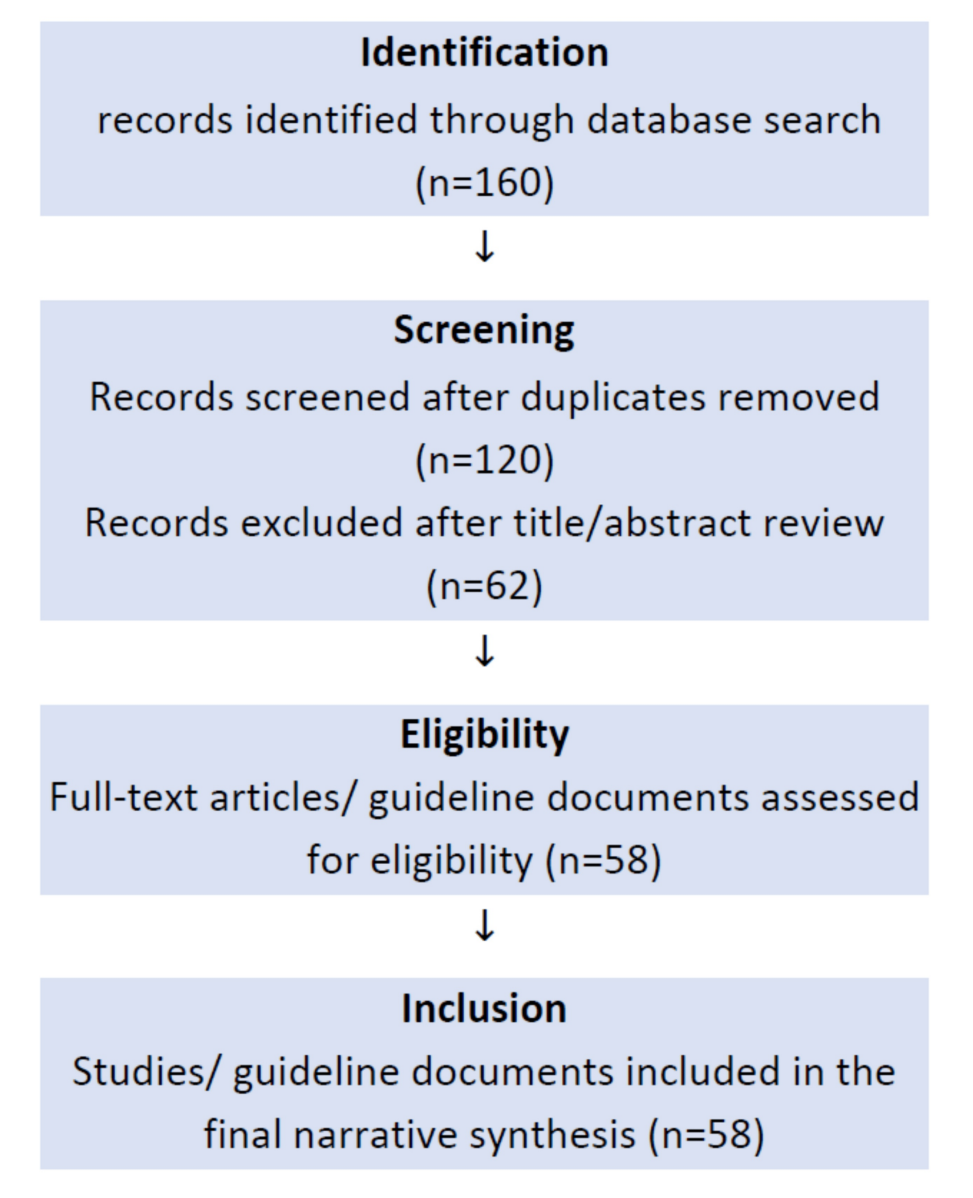
Literature selection process flowchart

This narrative review was developed through a targeted literature search of PubMed, Google Scholar, and key urological guideline repositories (EAU, AUA, NICE) between 1990 and 2025. Search terms included “digital rectal examination,” “prostate cancer diagnosis,” “DRE training,” and “clinical utility.” Articles were screened for relevance to diagnostic accuracy, clinical applications, and educational perspectives of DRE. Approximately 160 abstracts were screened, and 58 full-text articles and guidance documents were included based on relevance and quality (Figure 1). Both historical and contemporary sources were incorporated to reflect the evolution of DRE in clinical practice.

To ensure coherence and balance, the review was structured thematically, moving from diagnostic performance to educational, ethical, and global perspectives.

Detection with DRE in the era of PSA

Sensitivity and specificity of DRE for screening prostate cancer remain poor in both primary-care and referral populations. An old classical primary-care study has already demonstrated remarkable false negatives/positives, with inter-clinician differences [26]. A large systematic review conducted in a community screening environment (seven studies) reported that the pooled sensitivity was 0.51 and the pooled specificity was 0.59; they concluded that DRE alone is not appropriate for the asymptomatic men [11]. In comparison to PSA in meta-analysis, the adjunct of DRE is likely to improve specificity and with minimal or no benefit in sensitivity for clinically significant prostate cancer (csPCa) in contemporary settings [27]. In MRI-first pathways, an abnormal DRE with a normal PSA rarely identifies csPCa (single-digit/few yield), highlighting its limited independent utility for case-finding in asymptomatic men [28].

One of the statistical reasons for DRE’s observed performance fluctuation is dependent on pre-test probability: positive and negative predictive values vary by disease prevalence and with referral threshold. As discussed by Altman & Bland, predictive values can be influenced by the context; DRE’s positive predictive value will be lower in low-prevalence settings and higher when applied to ‘enriched’ patient cohorts [29].

Anatomical limitations of DRE

Anatomic limitations account for many “missed” cancers: anterior and apical lesions are commonly non-palpable despite clinical significance. Baseline studies comparing DRE to the location of pathologic findings demonstrated that a substantial portion of tumours were beyond the "reach" of DRE [30]. Even at low PSA levels, a significant fraction of biopsy-positive cancers exhibits normal DREs [31]. By contrast, in men who have already been marked out by elevated PSA, an abnormal DRE significantly increases the chance of biopsy-confirmed csPCa and can assist in stratification for early imaging/biopsy in resource-limited environments [32]. There is still a small subgroup of DRE suspicious tumours at low PSA; intriguingly, they may present with adverse pathologic features, highlighting the importance of paying heed to unequivocal abnormal exam even in the era of PSA [33].

Pathways for the diagnosis of prostate cancer by MRI and the role of DRE

Pre-biopsy work-up has been influenced by high-level evidence. The PROMIS trial showed that mpMRI is much more sensitive for csPCa than 12-core TRUS biopsy, and it may refer men to biopsy or avoid unnecessary biopsies [34]. PRECISION trial demonstrated MRI-targeted biopsy detected more csPCa and less indolent disease than systematic biopsy alone in biopsy-naïve patients [35]. Systematic + targeted biopsy combination after a positive MRI to maximise csPCa yield was supported by MRI-FIRST [36]. There are coherent MRI data synthesised in a Cochrane review, which unequivocally does confirm an increased diagnosis of csPCa with less biopsy oversampling if MRI is inserted into the pathway [37]. Multiparametric magnetic resonance imaging (mpMRI) is increasingly performed for the detection and risk stratification of clinically significant prostate cancer (csPCa), with ongoing needs for standardisation of techniques and training radiologists in its best practical use. To meet these challenges, the American College of Radiology (ACR) established a panel consisting of experts in prostate imaging from around the world to develop an updated version 2 of the Prostate Imaging Reporting and Data System (PI-RADS) [38]. The purpose of PI-RADS v2 is to standardise image-acquisition and prostate-MRI-reporting processes, and it is essential for patient management, as well as communication and research activity. PI-RADS v2 has been relatively quickly and broadly accepted by radiologists and urologists around the world, with its widespread incorporation in clinical practice and scientific work [38].

With MRI-first, the marginal diagnostic yield of routine DRE in asymptomatic men is still smaller, and many guidelines have therefore de-emphasised DRE for screening/initial case-finding but continued to support its use for symptom assessment and risk stratification [13-15].

Risk prediction and integrated models

DRE is poor as a single test, but its discrimination and specificity can be slightly improved by the addition of DRE findings to a multivariable risk calculator (for example, the PCPT and ERSPC tools) in an effort to reduce unnecessary biopsies at a given sensitivity. In the PCPT calculator, DRE was an independent predictor of risk and added to AUC gains by other risk factors within PSA strata [39]. There are also external/updated validations of ERSPC risk calculators that demonstrate additional specificity when DRE is added to the clinical covariates [40]. In reality, these models allow clinicians to down-weight an isolated borderline PSA if DRE is normal, or up-shift risk where DRE is clearly suspicious - as long as shared decision-making and MRI access inform the final step.

Biomarkers and DRE as complementary tools

Contemporary biomarkers, for example, PHI, 4Kscore, and PCA3, seek to enhance net benefit by more effectively distinguishing csPCa from indolent disease. The reviews outline their respective and overall performance compared to the reflex biopsy [41,42]. Crucially, if biomarkers do prove cost-effective when incorporated into multivariable models, a high-quality DRE may still provide the clinician with incremental pragmatic value as an inexpensive clinical cue (particularly whilst MRI availability is limited) but does not replace the need for mpMRI or high-quality biomarkers in the vast majority of clinical scenarios [41,42].

Patient acceptability, ethics, and professionalism

The DRE, one of the most intimate patient encounters in medical practice, has a substantial impact on uptake and diagnostic yield, influenced by patients' perception. Men may be reluctant to present early due to pain, embarrassment or cultural discomfort. Whilst over one-third of those in a community sample experienced moderate to severe embarrassment, information and emotional support significantly reduced anxiety, particularly in younger men or men with less formal education [43]. The DRE is not just the mechanics: clinicians are reminded that the DRE is more than simply a technical act, it is also a communicative event - how we "introduce," then explain and "conclude" it helps to preserve patient trust and satisfaction.

The GMC requires that clinicians discuss indications, offering a chaperone if patients wish and obtain informed consent for all intimate examinations in an environment that maintains dignity and privacy [44]. Just documenting this discussion explicitly - to the satisfaction of a court - will reassure patients and protect clinicians. These ethical doctrines pertain to inpatient, outpatient and teaching contexts and correspond with the professionalism demanded within contemporary UK practice.

Training, competency, and simulation-based learning

While still clinically relevant, opportunities to teach DRE safely and effectively remain sporadic. In a national survey, the majority of Irish medical graduates had performed the examination less than five times and lacked confidence and certainty with respect to technique and interpretation [45]. Time constraints, patient hesitance, and less bedside exposure are undermining the apprenticeship model that afforded frequent practice.

Higher education research now supports simulation and hybrid methods to allow repetition without loss of dignity. An augmented reality DRE simulator with haptic feedback and visualised anatomy has been shown to be feasible, acceptable and has objective force- and position-based metrics suited for deliberate practice with immediate debriefing [46]. In a similar context, a sensorised wearable glove for intimate pelvic or rectal examinations has been designed to continuously measure application pressure and keep track of exam duration in addition to trajectory, with an aim to provide quantitative feedback about insufficient and excessive force [47]. These advances allow for standardisation in technique and the establishment of objective competency criteria and align with present-day direction towards outcome-based medical education.

Clinical staging and oncologic context

Other than ultrasound, for established prostate cancer, DRE is still incorporated as a formal aspect of the clinical T-staging (cT) in the AJCC 8th Edition. Palpable asymmetry, capsular irregularity, or seminal-vesicle fixation is indicative of T2-T3 disease and differentiates organ-confined from locally advanced tumours [48]. Correct staging provides an aid for multidisciplinary decision-making, going forward to either nerve-sparing surgery, radiotherapy, or systemic therapy. While MRI is superior in spatial resolution for subtle extracapsular extension, DRE still provides immediate, direct and low-cost bedside information, which is particularly useful when imaging is not available or it may be equivocal.

Economic and pathway considerations

The introduction of MRI-first diagnostic pathways has revolutionised the work-up for prostate cancer, with increased detection rates for clinically significant tumours and a reduction in over-diagnosis of indolent disease. Cost-effectiveness analyses have shown that MRI-integrated pathways are cost-effective compared to biopsy-first strategies, due largely to reductions in unnecessary biopsies, procedure complications and subsequent overtreatment [49]. With regard to policy, a 2024 systematic review of European cost-effectiveness studies concluded that risk-adjusted screening strategies (based on PSA, MRI and targeted biopsy) provide greater health benefits per unit cost relative to universal-based screening among asymptomatic males. This is dependent on the age of the patients screened, with cost-effectiveness decreasing in older ages [50]. These results add further evidence that DRE is now best limited to a selective role in clinical assessment rather than being considered as a mass screening tool, with its role mainly being in triage and risk communication rather than population detection.

Global health and LMIC perspectives

Access barriers to MRI scan, PSA assay and specialist urology service in low- to middle-income countries (LMICs) prompt heavy dependence upon clinical examination. West-African multi-centre evaluation of the DRE combined with PSA was a far more useful adjunct than each method used individually [51]. Similarly, Ojewola et al. demonstrated that hardness, nodularity, or asymmetry on DRE remained independent predictors of disease after adjusting for the PSA level; thus, indicating the continued value for skilful clinical evaluation [52].

A subsequent trial from Nigeria supported that this approach increases case detection and preserves limited biopsy resources [53]. Such findings support that while DRE lacks the sensitivity for population-based screening, it is still a practical triage tool in resource-poor settings - a fact endorsed by regional professional bodies advocating cost-free methods of early detection.

Hospital clinical practice and patient experience

In the secondary care setting, DREs are conducted for a variety of reasons, ranging from bowel obstruction or rectal bleeding to pre-operative assessment. Qualitative evidence found that patients are willing to be examined if they feel that the examination is well justified and conducted in a professional manner; however, unclear or unjustified examinations create resentment and mistrust [54]. Communication, consent and chaperone use have also been incorporated into training programmes, which will lead to improvement in patient satisfaction and trainee confidence [55]. By inlaying these inter-personal skills within a procedural teaching framework, the DRE is no longer a traumatic ritual but becomes a respectful diagnostic encounter.

DRE for emergency neurological assessment and benign urological disease

Outside of oncology, the DRE is critical in the assessment of suspected cauda-equina syndrome, enabling bedside assessment of anal tone and perianal sensation pending imaging. One systematic review reported only moderate inter-observer reliability but stressed that abnormal findings, if combined with new urinary retention or saddle anaesthesia, would warrant emergency MRI and specialist referral [56].

In routine urological practice, DRE continues to hold a key position in the investigation of men suffering from LUTS. The European Association of Urology LUTS Guideline 2024 recommends the assessment of prostate size, consistency and tenderness to differentiate it from prostatitis or induration suspicious for malignancy [57]. In the UK, the NICE NG131 Guideline (2025) provides recommendations to use DRE during the first assessment and risk-stratification pathway [58], and assists in decision making by guiding PSA testing, imaging or urgent referral if findings are definitively abnormal.

Cumulatively, these recommendations validate that despite DRE stepping back from population screening, DRE remains as relevant today to day-to-day diagnostic reasoning in both oncological and benign presentations.

## Conclusions

DRE has evolved from a standalone investigation into a selective adjunct within a multimodal diagnostic environment. PSA, MRI and molecular markers now rule the landscape of early detection and staging; however, because it is immediate, inexpensive, available at bedside, and consequently not requiring any laboratory or advanced radiologic facilities, DRE still plays a key role in clinical pathways. Its shortcomings, especially poor sensitivity and inter-observer variation, are balanced by its usability in the evaluation of symptoms, staging of the disease and setting of global health issues where advanced imaging is scarce. Dignity and patient confidence during intimate examinations are contingent on ethical communication, informed consent, and the use of chaperones. From an educational perspective, standardised simulation and quantification have changed how DRE competence is trained for and evaluated, thereby guaranteeing the technical mastery of this procedure by tomorrow’s clinicians. Economic considerations DRE is a practical diagnostic triage for both resource-rich and resource-poor environments, permitting early identification of pathology before confirmatory testing.

In conclusion, the DRE continues to form the basis of physical examination and acts as an interface between traditional clinical practice and the present day. The enduring role in NICE, EAU and AUA guidelines emphasises its multi-dimensional applicability from oncological staging to neurological emergencies, confirming that as technology advances, clinical acumen based on direct examination remains a key aspect of modern medicine.
